# Rescue Mastectomy in a Patient With Locally Advanced Triple‐Negative Breast Cancer and Severe Biochemical Hypothyroidism: A Multidisciplinary Ethical and Surgical Dilemma

**DOI:** 10.1155/crom/3822467

**Published:** 2026-04-19

**Authors:** Nelson Buelvas

**Affiliations:** ^1^ Department of Surgery, Universidad del Sinú (University of Sinu), Cartagena, Colombia; ^2^ IMAT Oncomédica–Auna (Advanced Technology Medical Institute), Montería, Colombia

**Keywords:** mastectomy, multidisciplinary decision-making, rescue surgery, severe hypothyroidism, triple-negative breast cancer

## Abstract

Severe hypothyroidism is traditionally considered a contraindication to elective major surgery due to the risk of perioperative metabolic decompensation and myxedema coma. However, in oncologic settings, delaying surgery may result in irreversible loss of resectability and adverse outcomes. Evidence guiding surgical decision‐making in patients with advanced cancer and severe endocrine dysfunction remains limited. We report the case of a patient with triple‐negative breast cancer initially staged as IIA who discontinued neoadjuvant chemotherapy and subsequently developed rapid locoregional progression to stage IIIB. Despite reinduction with chemotherapy and immunotherapy, the tumor remained refractory, and rescue mastectomy was indicated as the only remaining oncologic option. Preoperative assessment unexpectedly revealed severe biochemical hypothyroidism with markedly elevated thyroid‐stimulating hormone levels and reduced free thyroxine, raising concern for perioperative metabolic decompensation and myxedema coma. Given the risk of permanent loss of operability associated with surgical delay and the prolonged time required to achieve full biochemical euthyroidism, a multidisciplinary decision was made to proceed with surgery after partial endocrine optimization using oral levothyroxine. Following extensive informed consent and anesthetic planning, rescue mastectomy with wide elliptical skin excision was performed without intraoperative or postoperative complications. The patient had an uneventful recovery, with negative surgical margins and no need for postoperative ventilatory support. This case highlights the challenges of managing severe biochemical hypothyroidism in time‐sensitive oncologic surgery. When surgery represents the only viable therapeutic option, individualized multidisciplinary decision‐making and shared risk assessment may justify proceeding despite incomplete metabolic optimization. Rescue surgery may be considered in carefully selected high‐risk patients when the oncologic benefit outweighs the potential perioperative risk.

**Learning Points**

• Severe hypothyroidism may be clinically silent despite extreme biochemical abnormalities.
• Perioperative guidelines for hypothyroidism are largely based on nononcologic settings and may not apply to time‐sensitive cancer cases.
• Delaying surgery in locally advanced, refractory breast cancer may result in irreversible loss of resectability.
• Multidisciplinary discussion and shared decision‐making are essential in high‐risk surgical scenarios.
• Breast surgeons should be familiar with less commonly used mastectomy designs to manage complex rescue cases.

## 1. Introduction

Triple‐negative breast cancer (TNBC) is an aggressive subtype characterized by rapid growth, early recurrence, and limited therapeutic targets [1]. Optimal outcomes depend on strict adherence to multimodal treatment strategies, including timely systemic therapy and surgery [2]. Disruptions in treatment continuity may result in rapid disease progression and loss of curative opportunities.

Rescue mastectomy represents a controversial but sometimes indispensable option in patients with locally advanced breast cancer who fail systemic therapy [3]. In such cases, surgical delay may result in permanent loss of operability and increased risk of locoregional and systemic progression. The decision to proceed with surgery is further complicated when severe comorbidities are present.

Severe hypothyroidism is classically considered a contraindication to major elective surgery due to its association with cardiovascular instability, respiratory depression, altered drug metabolism, and the risk of myxedema coma [4–6]. Current perioperative guidelines, however, are largely derived from nononcologic scenarios and provide limited guidance for time‐sensitive cancer cases [7, 8]. We report a case illustrating this dilemma and highlight the role of individualized, multidisciplinary surgical decision‐making.

## 2. Case Presentation

A middle‐aged woman was diagnosed in September 2024 with right‐sided breast cancer, initially staged as IIA (cT2N0M0), with a triple‐negative molecular profile. She received four cycles of neoadjuvant anthracycline–cyclophosphamide chemotherapy, completed in November 2024 at an outside institution. After this, the patient discontinued oncologic follow‐up at the initial treating institution and pursued alternative therapies, including apitherapy, leading to rapid locoregional progression with skin ulceration.

**Figure 1:**
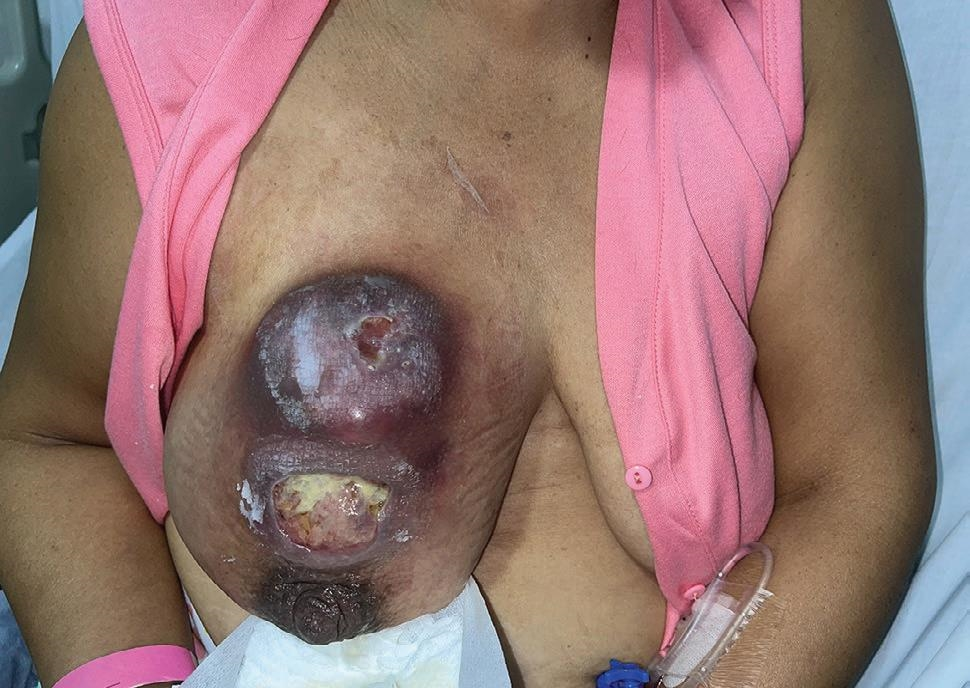
Clinical presentation at initial surgical consultation showing a locally advanced right breast tumor with marked skin infiltration and ulceration, consistent with cT4b disease.

Upon presentation to our comprehensive cancer center in 2025, the tumor was restaged as IIIB (cT4bN0M0). Neoadjuvant chemotherapy with carboplatin and taxanes combined with immunotherapy (pembrolizumab) was initiated. After three cycles, the patient showed clear local progression (Figure 1), and further systemic therapy was considered unlikely to achieve meaningful tumor regression. Rescue mastectomy was therefore indicated as the only remaining oncologic option to achieve local control and prevent further progression to unresectability.

Preoperative anesthetic evaluation on August 15, 2025, unexpectedly revealed severe biochemical hypothyroidism, with a thyroid‐stimulating hormone (TSH) level of 103.1 *μ*IU/mL (reference range: 0.35–4.94 *μ*IU/mL) and a free thyroxine (FT4) level of 0.4 ng/dL. The patient had minimal clinical manifestations despite the severity of the biochemical hypothyroidism. The patient had no known prior history of thyroid disease, no previous use of thyroid medication, and no history of neck irradiation or endocrine comorbidities. Before the diagnosis of breast cancer, she had been considered previously healthy. Given the narrow oncologic window, she was admitted for inpatient endocrine optimization and close monitoring. Intravenous levothyroxine was not available in our institution at the time of hospitalization. Given the patient′s clinical stability and absence of features suggestive of myxedema coma, oral levothyroxine therapy was initiated with close inpatient monitoring in consultation with internal medicine and endocrinology. Oral levothyroxine was initiated at 150 *μ*g daily. Serial laboratory measurements demonstrated a gradual increase in FT4 over the following days, while TSH remained markedly elevated. On August 26, 2025, the levothyroxine dose was increased to 175 *μ*g daily, with recognition that complete biochemical euthyroidism would require several weeks to months. Serial thyroid function tests demonstrated progressive improvement in free thyroxine levels following initiation of levothyroxine therapy (Table 1), although TSH levels remained markedly elevated.

**Table 1:** Serial thyroid function tests during hospitalization (“—” indicates test not performed that day). Surgery was performed on August 28, 2025.

Date	FT4 (ng/dL)	TSH (*μ*IU/mL)
August 15, 2025	0.4	103.1
August 18, 2025	0.5	—
August 21, 2025	0.65	—
August 22, 2025	0.67	—
August 24, 2025	0.72	98.4
August 25, 2025	0.70	61.2

After multidisciplinary tumor board discussion involving surgical oncology, anesthesiology, internal medicine, and endocrinology, and following extensive informed consent addressing the elevated risk of perioperative metabolic decompensation and potential myxedema coma, the decision was made to proceed with surgery during the same hospitalization. An intensive care unit bed was reserved preoperatively.

**Figure 2:**
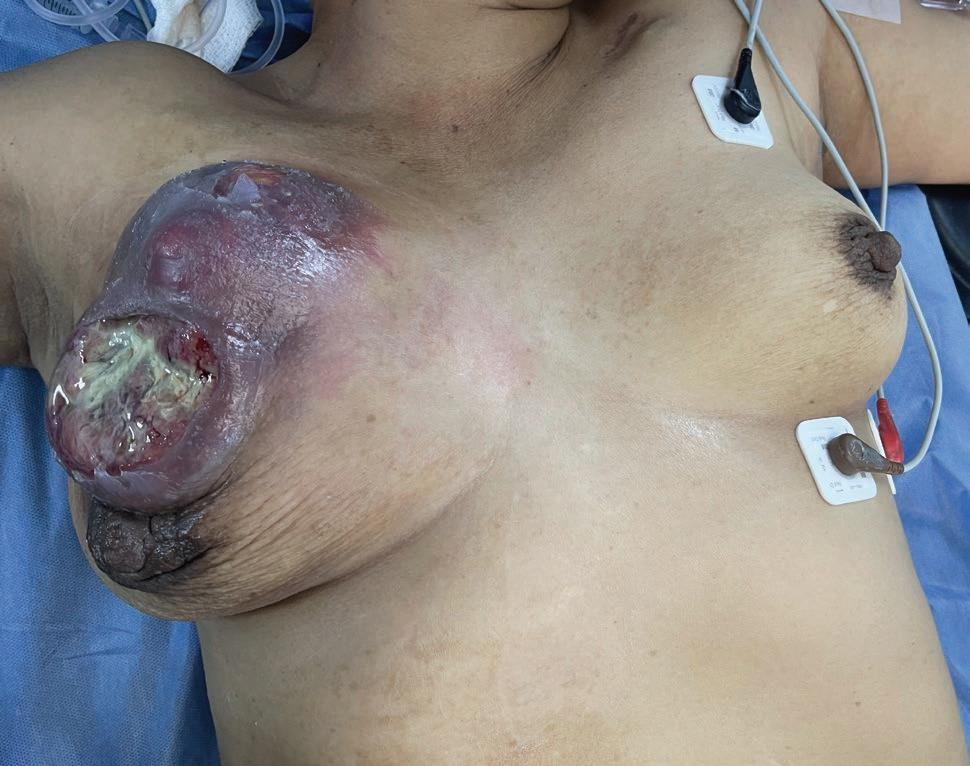
Immediate preoperative appearance of the right breast demonstrating extensive tumor burden with progressive skin involvement and ulceration prior to rescue mastectomy.

**Figure 3:**
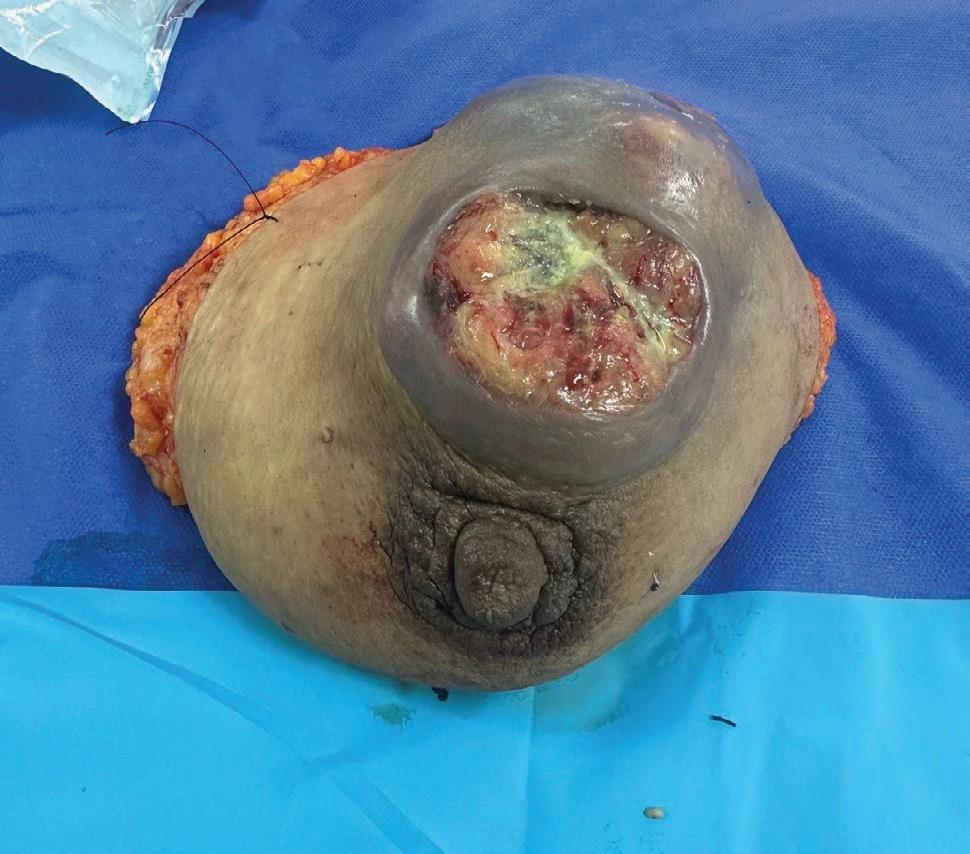
Surgical specimen from the rescue mastectomy, illustrating the large tumor mass with overlying ulcerated skin and the extent of resection required to achieve adequate oncologic margins.

On August 28, 2025, the patient underwent rescue mastectomy (Figure 2). A wide elliptical skin incision was designed to accommodate the predominantly vertical cephalocaudal distribution of the tumor and the extensive area of skin involvement (Figure 3). The procedure consisted of a total mastectomy with axillary lymph node dissection. The pectoralis major and minor muscles were preserved, as there was no evidence of direct muscular invasion. The incision design resembled the classic vertical elliptical approach historically described in early mastectomy techniques. Given the severity of the biochemical hypothyroidism, particular attention was paid to perioperative anesthetic management. General anesthesia was administered with careful monitoring due to the potential risks of hypoventilation, hypothermia, and hemodynamic instability associated with severe hypothyroidism. Temperature control and hemodynamic stability were maintained throughout the procedure. The patient remained intraoperatively stable and did not require vasopressor support. The patient had an uneventful postoperative course and was discharged on August 30, 2025.

Final pathology revealed an invasive carcinoma of no special type (NST), Grade 3, measuring 16 × 8 × 7 cm, with associated high‐grade ductal carcinoma in situ showing cribriform, solid, and comedo patterns. Necrosis involved approximately 30% of the specimen. The overlying skin was ulcerated, consistent with the clinical T4b classification. All peripheral and deep surgical margins were free of tumor. Seventeen axillary lymph nodes were dissected, all negative for metastasis (0/17). Lymphovascular and perineural invasion were absent.

**Figure 4:**
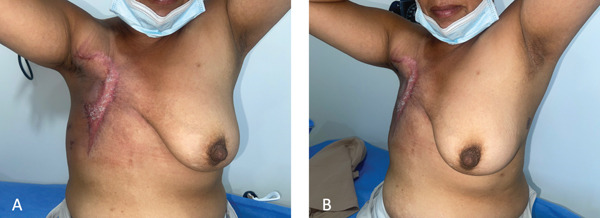
Postoperative outcome following rescue mastectomy. (A) Frontal view showing adequate wound healing and chest wall coverage. (B) Lateral view demonstrating satisfactory contour and absence of early postoperative complications.

During follow‐up, germline genetic testing identified a pathogenic heterozygous BRCA1 mutation, consistent with the patient′s strong family history of breast cancer. She subsequently completed adjuvant radiotherapy to the chest wall using intensity‐modulated radiotherapy (IMRT), delivering a total dose of 40 Gy in 15 fractions, with partial use of bolus according to the radiation treatment plan. She remains under multidisciplinary oncologic follow‐up (Figure 4).

## 3. Discussion

Locally advanced TNBC is characterized by aggressive biological behavior and rapid locoregional progression [1]. In cases with extensive skin involvement or ulceration, continued tumor growth may lead to chest wall invasion, worsening ulceration, infection, or systemic deterioration, which can ultimately compromise surgical resectability. Therefore, prolonged surgical delay while awaiting full biochemical normalization of thyroid function posed a significant oncologic risk in this patient. In this context, the multidisciplinary team considered that the potential oncologic harm associated with prolonged surgical delay outweighed the metabolic risks associated with operating under partially corrected hypothyroidism.

Rescue mastectomy remains a controversial but sometimes necessary intervention in such scenarios [3]. Although not always curative, surgery may represent the only opportunity to achieve local disease control and prevent irreversible loss of operability [3, 9]. Therefore, prolonged surgical delay while awaiting complete biochemical normalization of thyroid function posed a significant oncologic risk in this patient. Delaying surgery in patients with progressive, skin‐infiltrating tumors may result not only in technical inoperability but also in ongoing tumor burden with increased risk of locoregional complications and potential systemic progression [10].

Current endocrine guidelines generally recommend postponing elective surgery in patients with severe hypothyroidism because of increased perioperative risks, including hypotension, hypothermia, respiratory depression, and in extreme cases myxedema coma [4–6]. However, these recommendations are largely derived from nononcologic settings and provide limited guidance in situations in which delaying surgery may result in significant oncologic harm [7, 8, 11, 12]. In this case, severe biochemical hypothyroidism was incidentally detected during preoperative evaluation, despite minimal clinical manifestations, highlighting the potential dissociation between laboratory severity and clinical presentation.

Although the primary focus of this report is surgical decision‐making, it is noteworthy that immune checkpoint inhibitors are associated with endocrine adverse events, including hypothyroidism [13]. Although a definitive causal relationship could not be established, treatment‐related thyroid dysfunction cannot be excluded. Importantly, acknowledging this possibility does not imply causation or fault, but rather underscores the importance of multidisciplinary vigilance.

This case posed a genuine ethical dilemma: Proceeding with surgery entailed a substantial metabolic risk, whereas delaying surgery to achieve biochemical euthyroidism would likely have resulted in definitive loss of resectability. The decision to operate was therefore based on individualized risk–benefit assessment, multidisciplinary consensus, and shared decision‐making with the patient.

From a technical standpoint, a wide vertical‐elliptical skin incision was selected over more conventional incision patterns, such as Stewart or Orr designs, which would not have permitted adequate oncologic skin sacrifice. This highlights the importance for breast surgeons to remain familiar with less commonly employed surgical designs to manage atypical tumor topographies in rescue scenarios.

## 4. Limitations

As a single case report, definitive causal relationships cannot be established, and long‐term oncologic outcomes remain unknown. Additionally, full biochemical correction of hypothyroidism prior to surgery was not achievable within the available oncologic timeframe. Nevertheless, this case provides valuable insight into real‐world decision‐making when standard perioperative recommendations conflict with oncologic urgency.

## 5. Conclusion

Severe biochemical hypothyroidism may be clinically silent yet pose significant perioperative risks. In selected patients with time‐sensitive oncologic indications, rescue surgery may be justified despite incomplete metabolic optimization when guided by multidisciplinary evaluation, thorough preoperative planning, and shared decision‐making. This case supports an individualized approach to surgical decision‐making in complex oncologic emergencies.

## Funding

No funding was received for this manuscript.

## Ethics Statement

Written informed consent for publication of clinical details and images was obtained from the patient.

## Conflicts of Interest

The author declares no conflicts of interest.

## Data Availability

Data sharing is not applicable to this article as no datasets were generated or analyzed during the current study.

## References

[1] Dent R. , Trudeau M. , Pritchard K. I. , Hanna W. M. , Kahn H. K. , Sawka C. A. , Lickley L. A. , Rawlinson E. , Sun P. , and Narod S. A. , Triple-Negative Breast Cancer: Clinical Features and Patterns of Recurrence, Clinical Cancer Research. (2007) 13, no. 15, 4429–4434, 10.1158/1078-0432.CCR-06-3045, 2-s2.0-34547661993. 17671126

[2] Bianchini G. , Balko J. M. , Mayer I. A. , Sanders M. E. , and Gianni L. , Triple-Negative Breast Cancer: Challenges and Opportunities of a Heterogeneous Disease, Nature Reviews Clinical Oncology. (2016) 13, no. 11, 674–690, 10.1038/nrclinonc.2016.66, 2-s2.0-84994112876, 27184417. PMC5461122 27184417

[3] NCCN , Clinical Practice Guidelines in Oncology: Breast Cancer, Version 5.2025, 2025, National Comprehensive Cancer Network, Accessed October 16, 2025.

[4] Palace M. R. , Perioperative Management of Thyroid Dysfunction, Health Services Insights. (2017) 10, no. 10, 1178632916689677, 10.1177/1178632916689677. 28469454 PMC5398303

[5] Ladenson P. W. and Kim M. I. , Perioperative Management of Patients With Hypothyroidism, Endocrinology and Metabolism Clinics of North America. (2003) 32, no. 2, 503–518, 10.1016/S0889-8529(03)00007-0, 2-s2.0-0038320299. 12800543

[6] Klein I. and Ojamaa K. , Thyroid Hormone and the Cardiovascular System, New England Journal of Medicine. (2001) 344, no. 7, 501–509, 10.1056/NEJM200102153440707, 2-s2.0-0035864975. 11172193

[7] Himes C. P. , Ganesh R. , Wight E. C. , Simha V. , and Liebow M. , Perioperative Evaluation and Management of Endocrine Disorders, Mayo Clinic Proceedings. (2020) 95, no. 12, 2760–2774, 10.1016/j.mayocp.2020.05.004, 33168157. 33168157

[8] Malhotra B. and Bhadada S. K. , Perioperative Management for Non-Thyroidal Surgery in Thyroid Dysfunction, Indian Journal of Endocrinology and Metabolism. (2022) 26, no. 5, 428–434, 10.4103/ijem.ijem_273_22, 36618525. 36618525 PMC9815191

[9] Xie X. , Li H. , Wang C. , Li W. , Xie D. , Li M. , and Jiang D. , Effect of Modified Radical Mastectomy Combined With Neo-Adjuvant Chemotherapy on Postoperative Recurrence Rate, Negative Emotion, and Life Quality of Patients With Breast Cancer, American Journal of Translational Research. (2022) 14, no. 1, 460–467, 35173865. 35173865 PMC8829650

[10] Bleicher R. J. , Timing and Delays in Breast Cancer Evaluation and Treatment, Annals of Surgical Oncology. (2018) 25, no. 10, 2829–2838, 10.1245/s10434-018-6615-2, 2-s2.0-85049569835, 29968031. 29968031 PMC6123282

[11] Jonklaas J. , Bianco A. C. , Bauer A. J. , Burman K. D. , Cappola A. R. , Celi F. S. , Cooper D. S. , Kim B. W. , Peeters R. P. , Rosenthal M. S. , Sawka A. M. , and American Thyroid Association Task Force on Thyroid Hormone Replacement , Guidelines for the Treatment of Hypothyroidism: Prepared by the American Thyroid Association Task Force on Thyroid Hormone Replacement, Thyroid. (2014) 24, no. 12, 1670–1751, 10.1089/thy.2014.0028, 2-s2.0-84918583260, 25266247. 25266247 PMC4267409

[12] Zhang Y. , Chu L. , and Han H. , Myxedema Coma: Challenges and Future Directions, a Systematic Survey and Review, Thyroid Research. (2025) 18, no. 1, 10.1186/s13044-025-00268-1, 41053871. PMC12502585 41053871

[13] Barbesino G. , Immune Checkpoint Inhibitor–Induced Thyroid Dysfunction, Journal of Clinical Endocrinology & Metabolism. (2020) 105, dgz286, 10.1210/clinem/dgz286, 31853158. 32668461

